# Twitter and Non-Elites: Interpreting Power Dynamics in the Life Story of the (#)BRCA Twitter Stream

**DOI:** 10.1177/2056305117733224

**Published:** 2017-09-23

**Authors:** Stefania Vicari

**Affiliations:** The University of Sheffield, UK

**Keywords:** Angelina Jolie, BRCA, broadcasting, framing, gatekeeping, Twitter

## Abstract

In May 2013 and March 2015, actress Angelina Jolie wrote in the *New York Times* about her choice to undergo preventive surgery. In her two op-eds, she explained that − as a carrier of the BRCA1 gene mutation − preventive surgery was the best way to lower her heightened risk of developing breast and ovarian cancer. By applying a digital methods approach to BRCA-related tweets from 2013 and 2015, before, during, and after the exposure of Jolie’s story, this study maps and interprets Twitter discursive dynamics at two time points of the BRCA Twitter stream. Findings show an evolution in curation and framing dynamics occurring between 2013 and 2015, with individual patient advocates replacing advocacy organizations as top curators of BRCA content and coming to prominence as providers of specialist illness narratives. These results suggest that between 2013 and 2015, Twitter went from functioning primarily as an organization-centered news reporting mechanism, to working as a crowdsourced specialist awareness system. This article advances a twofold contribution. First, it points at Twitter’s fluid functionality for an issue public and suggests that by looking at the life story—rather than at a single time point—of an issue-based Twitter stream, we can track the evolution of power roles underlying discursive practices and better interpret the emergence of non-elite actors in the public arena. Second, the study provides evidence of the rise of activist cultures that rely on fluid, non-elite, collective, and individual social media engagement.

## Introduction

On 14 May 2013, actress Angelina Jolie wrote a *New York Times* opinion piece to explain her choice to undergo prophylactic double mastectomy. As a carrier of the rare BRCA1 gene mutation—Jolie explained—preventive surgery had lowered her heightened risk of developing breast cancer.^1^ Following the op-ed, the story was picked up by news and entertainment media in both Western and Asian periodicals, with the celebrity’s picture appearing on the covers of *People* and *Time* magazines and the story remaining of news value for months (Borzekowski, Guan, Smith, Erby, & Roter, 2014; Evans et al., 2014; Kamenova, Reshef, & Caulfield, 2014; Noar, Althouse, Ayers, Francis, & Ribisi, 2015). Two years later, on 24 March 2015, Jolie wrote about her second preventive surgery: the removal of ovaries and fallopian tubes.

Jolie’s story was given primarily positive offline (Kamenova et al., 2014) and online (Dean, 2016) institutional media coverage, with however little attention being drawn to the rarity of her genetic condition (Borzekowski et al., 2014). Following Jolie’s first op-ed, a so-called “Angelina effect” (Kluger et al., 2013) was measurable in the steady increase in requests for genetic testing (Barton, 2013; Dunlop, Kirk, & Tucker, 2014; Evans et al., 2014; Kosenko, Binder, & Hurley, 2016; Mehta, 2013; Neporent, 2013) and relevant online information seeking (Noar et al., 2015).

This study turns to Twitter as a “networked space” (Jackson & Foucault Welles, 2016) where citizen-led or citizen-participated discussion contributes to the construction of knowledge around specific issues. Previous research showed that Twitter’s socio-technical infrastructure enables the coming to prominence of individual, non-elite actors, who engage in successful curation and framing dynamics (Hermida, 2015; Jackson & Foucault Welles, 2015, 2016; Meraz & Papacharissi, 2013). However, the shape of these dynamics—and their effect on power structures—at different time points of an issue-based Twitter stream remains unexplored.

The BRCA Twitter stream provides a relevant case study to test the impact of temporality on both the power dynamics underlying Twitter’s discursive work and Twitter’s overall functionality for issue publics. Jolie’s announcements in May 2013 and March 2015 created similar media ecological conditions, with mainstream media exposure of and heightened public debate around BRCA-related topics. By mapping, interpreting, and comparing curation and framing dynamics in the BRCA Twitter stream around both publication dates, this article advances a twofold contribution. First, it points at Twitter’s fluid functionality for issue publics and provides original insight into the need to look at the life story of issue-based Twitter streams to fully understand the changing role of social media platforms in enhancing old and new power structures underlying discursive practices. Second, it provides evidence of the emergence of activist cultures—in this case of health and illness—that rely on non-elite collective and individual social media engagement.

## Twitter and Crowdsourced Discourse

As the most popular Western microblogging platform, Twitter provides a space where information is produced, shared, amplified, or quickly lost. Researchers have drawn attention to the way Twitter has created new ways to organize shared knowledge, where individual and collective actors participate in the development of collaborative information and mobilization dynamics. But how do these different actors engage in social media discursive practices? Exploring contemporary instances of activism, Bennett and Segerberg (2013) distinguish between two new forms of digitally driven-civic engagement: “organizationally enabled” and “crowd-enabled” “connective action.” In organizationally enabled connective action, the availability of social media platforms—interpreted by the authors as “digitally networking mechanisms”—allows organizations to mobilize individuals around issues of interest. In crowd-enabled connective action, individuals use digitally networking mechanisms to join activist campaigns without the intermediation of traditional organizations. The logic of connective action draws upon the idea that digital media in general − and social media platforms in particular − allow the emergence of collaborative processes based on shared personal frames of action.

In the attempt to describe the nature of these collaborative processes, Hermida (2010) defines Twitter’s functionality as that of a “collective intelligence” and an “awareness system”^2^ (p. 298)—that is, one that allows users to incorporate information and knowledge deriving from varied sources, challenging traditional protocols of public communication. In other words, the idea of a Twitter-generated “awareness system” backgrounds questions of information reliability while foregrounding issues of knowledge inclusivity. In fact, Twitter does not necessarily enhance intentionally collaborative processes (Kwak, Lee, Park, & Moon, 2010; Vicari, 2013), but it does show potential for broadcasting and gatekeeping of user-generated or user-selected content (Bastos, Galdini Raimundo, & Travitzki, 2013; Benkler, 2006, p. 271; Boyd, Golder, & Lotan, 2010; Bruns & Burgess, 2012; Jackson & Foucault Welles, 2015, 2016; Meraz & Papacharissi, 2013; Tremayne, 2014). This happens via mechanisms that are platform-bound, that is, mechanisms that express themselves via the use of Twitter conversational (i.e., @, RT, and via) and tagging (i.e., #) markers.

Researchers have also drawn attention to the way these crowdsourced dynamics happen within a “hybrid media system” (Chadwick, 2013), one where “a global integration of different types and systems of media—personal and mass, national and international” (Bennett, Segerberg, & Walker, 2014, p. 232)—sees content bouncing back and forth between mainstream and social media. In fact, Papacharissi and de Fatima Oliveira (2012) have defined Twitter’s functionality as that of a “news reporting mechanism,” or one that allows the integration of different forms of news reporting. The “ambient” aspect of Twitter (Bruns & Burgess, 2012; Hermida, 2010) reveals itself exactly in this overlapping of different levels of communication and media practices: from background, always on, “mundane and phatic” posting, to sudden shifts in vocabulary, topic, tone, and targets when important news enters the Twittersphere (Bruns & Burgess, 2012, p. 802). Not only does this happen in second-screen (Giglietto & Selva, 2014; Iannelli & Giglietto, 2014) or dual screening (Vaccari, Chadwick, & O’Loughlin, 2015) practices—where social media users live-comment mainstream media content. Research has shown that this also occurs, for instance, in activist dynamics (Bennett et al., 2014; Jackson & Foucault Welles, 2015) and in microblogging about events of public interest (Papacharissi and de Fatima Oliveira, 2012; Vicari, 2013).

To investigate the shape of discursive work on Twitter, we then need to focus on two dimensions that enhance both crowdsourcing and hybrid dynamics: (1) the curation of content produced and shared in discursive streams and (2) the construction of meaning around the issues at the core of these streams.

## Broadcasting and Gatekeeping: The Networked Curation of Twitter Content

Twitter discursive work expresses itself in the constant live-streaming of 140-character posts where conversational markers can facilitate networking dynamics among users. Retweeting implies exposing someone else’s tweet to a wider public; via is a way to pay tribute to a source and @ is a conversational marker that allows users to mention or direct posts to other users, sustaining “a high level of interactivity and engagement” (Meraz & Papacharissi, 2013, p. 140). In the ecology of live-streaming, these three mechanisms entail drawing attention to a set of privileged Twitter users, more specifically, to their username (by using RT, via, or @) and to content produced (via) or tweeted (RT) by them. Those who engage in RT, via, or @ use can then be seen as broadcasters as they amplify other users’ voice and/or identity. Bennett et al. (2014) define these mechanisms as part of the broader process of “curation” that entails “the preservation, maintenance, and sorting of digital assets” (p. 239).

Drawing upon work by Smith, Rainie, Shneiderman, and Himelboim (2014), Jackson and Foucault Welles (2015, 2016) add to the understanding of Twitter broadcasting dynamics by describing #myNYPD and #Ferguson Twitter streams as “broadcast networks.” Broadcast networks have “a distinctive hub-and-spoke structure where most nodes in the network radiate out from a small number of central nodes” (Jackson and Foucault Welles, 2015, p. 938). According to the authors, Twitter broadcast networks—by potentially elevating to prominence any of their elite or non-elite nodes—generate conversational space for “minority viewpoints” otherwise missing in mainstream public sphere dynamics. Jackson and Foucault Welles go as far as to define Twitter broadcast networks as “networked counterpublics,” that is, the ultimate, networked expression of Fraser’s (1990) “parallel discursive arenas where members of subordinated social groups invent and circulate counterdiscourses, which in turn permit them to formulate oppositional interpretations of their identities, interests, and needs” (p. 67).

In an issue-based Twitter stream—or the collection of tweets relevant to a well-defined issue—while broadcasters extend the life-span of successful Twitter content, gatekeepers are Twitter users crowdsourced to prominence via the use of conversational markers. In other words, Twitter users whose messages are most frequently retweeted or shared with the via conversational marker or whose handle is most frequently mentioned via the @ marker become the gatekeepers of an issue-based Twitter stream. As Bastos et al. (2013, p. 3) suggest, “Gatekeeping is still a key mechanism in digital networks, only now it has been redesigned to incorporate a multitude of senders and receivers.” In fact, by highlighting the networked nature of discursive dynamics on social media platforms, the concept of “networked gatekeeping” (Meraz & Papacharissi, 2013) underlines the potential turning of traditionally non-elite actors into primary sources of information.

Hence, the investigation of platform-bound curation practices provides insight into the power relations underlying discursive work on Twitter. However, to understand the construction of meaning in issue-based Twitter streams, we need to focus on the actual content produced, shared, and/or dismissed via these curation practices.

## Frame Articulation via Hashtag Use

While users differently participate in Twitter’s awareness system, this system itself develops around the emergence of dominant narratives driven by successful hashtags. In fact, “In scenarios where communities converge on a selected hashtag to represent an issue or topic, such hashtags aid in the creation of an ad hoc issue public, collating tweets along a specific, topical dimension” (Meraz & Papacharissi, 2013, p. 143). If hashtags play such a central role in the emergence of dominant narratives—and ad hoc issue publics—we can approach them as “framing devices” (Gamson & Modigliani, 1989), or semantic elements used to convey specific frames around selected issues.

Drawing upon Bateson’s (1972) and Goffman’s (1974) early work, scholars across the social sciences have used the concept of frame to explain interpretative frameworks developed by individual and collective actors “for making sense of relevant events” (Gamson & Modigliani, 1989, p. 3). More specifically, frames are “schemata of interpretation” (Goffman 1974, p. 21) applied to any element of social reality; they “help internalize past experience and guide future action/reaction to upcoming events” (Vicari, 2010, p. 506).^3^

Meraz and Papacharissi (2013) have applied frame theory to the analysis of Twitter hashtags using frame concepts to explore, map, and interpret prominent hashtags during the 2011 Egypt uprisings. Their day-by-day investigation shows that quantitatively prominent hashtags—and hence dominant narratives—converged around the most significant events occurring during the protests. In their words, “the crowd converged on the framing of the event through reference to content-based frames such as key dates, geographic locations, and political figures” (p. 153).

Dahlberg-Grundberg and Lindgren (2014) looked at the use of tweets with multiple hashtags as an indication of “frame articulation” and “frame alignment,” that is, the drawing of relationships between previously disconnected issues. In their study of the Canadian Idlenomore movement, the authors suggest that the use of the movement’s #idlenomore hashtag with other movement hashtags (e.g., #ows for Occupy Wall Street and #Egypt for the 2011 Egypt uprising) shows “some form of symbolic entanglement of movements that is facilitated by the connective structures and the communicational affordances that the Twitter platform offers” (p. 22). The authors conclude that social media seem to enable a “networked form of crowdsourcing” in the development of dominant social movement narratives (p. 70).

Overall, research applying frame theory to the analysis of issue-based Twitter streams focuses on the discursive dimension of meaning construction, overlooking tweeters’ “attachment” to the issues they publicize and to Twitter itself (Marres, 2007). In other words, they tend to skim over Twitter users’ personal involvement with the issue being discussed and their familiarity with the platform where the discussion is taking place. What this research does show, however, is that hashtags can be successfully investigated as symbolic devices enabling both the emergence of specific representations of reality and the articulation of meaning along topical dimensions. What remains unclear is, however, how variations in hashtag use at different time points of a Twitter stream may suggest variations in frame articulation, discursive practices, and, ultimately, Twitter functionality for issue publics.

## Research Questions

By drawing upon findings on curation and framing dynamics, previous research has described Twitter as a platform enhancing the emergence of traditionally marginalized voices or non-elite actors and original representations of reality (Bastos et al., 2013; Hermida, 2015; Jackson & Foucault Welles, 2016; Meraz & Papacharissi, 2013). However, in most cases, while providing insight into Twitter’s functionality (e.g., “awareness system,” “news reporting mechanism,” “digitally networking mechanism”) at a crucial turn in the life story of an issue-based Twitter stream, this research fails to investigate the stability of the power roles, framing dynamics and ultimately platform functionality that it uncovers. To fill this gap, we need to incorporate a temporal dimension in the study of discursive practices and compare curation and framing dynamics at different time points of an issue-based Twitter stream.

The BRCA Twitter stream offers a significant case study to integrate temporality in the investigation of power dynamics in Twitter discursive practices. First, contemporary health communication research has highlighted the development of new active forms of patient engagement where patients—traditionally non-elite actors—often directly engage in the production of health knowledge (Vicari & Cappai, 2016). A health issue–based Twitter stream makes then for a relevant case study to analyze the emergence of non-elites in discursive work. Second, Jolie’s announcement in March 2015 reproduced similar media ecological conditions to those of May 2013—when her first op-ed was published—with heightened mainstream media exposure of BRCA-related topics. By mapping discursive practices in the BRCA Twitter stream around both publication dates, we can compare actors’ power roles and the articulation of meaning around the BRCA gene mutation at two similar—but temporarily distant—crucial points in the life story of the BRCA stream. More specifically, this study is addressing the following research questions:

*RQ1*. How do actor power relations (i.e., broadcasting and gatekeeping dynamics) develop through different crucial points in the life story of an issue-based Twitter stream?
*RQ2*. How do prominent frames develop through different crucial points in the life story of an issue-based Twitter stream?
*RQ3*. Do shifts in power relations and meaning construction at different crucial points in the life story of an issue-based Twitter stream suggest changing patterns in Twitter’s platform functionality (e.g., “awareness system,” “news reporting mechanism,” “digitally networking mechanism”) for an issue public?

## Data, Sample, and Methods

To scrape Twitter data relevant to the BRCA gene mutation around the publication of Jolie’s op-eds, I focused on tweets posted over two sample periods: 14 April–13 June 2013 and 24 February–23 April 2015, that is, each starting 1 month before the publication of each Jolie’s op-ed and ending 1 month after it. Using the Discovertext Sifter application—which relies on GNIP service for firehose access to Twitter—I launched two historical searches based on the following queries: “brca since:14-04-2013 until:13/06/2013” and “brca since: 24-02-2015 until:23-04-2015.” Non-BRCA-related tweets and tweets in a language different from English were then excluded from the results.^4^ Hence, this study investigates two Twitter archives—from 2013 and 2015—populated by 25,400 and 18,909 tweets, respectively.

**Figure 1.**
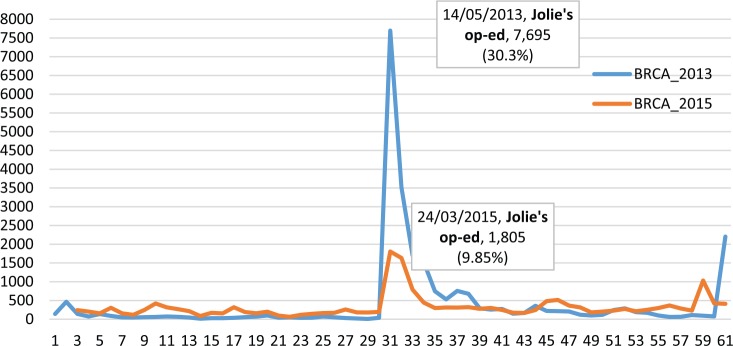
BRCA-related Twitter activity over the 2013 and the 2015 sample periods.

Figure 1 compares activity levels from Days 1 to 61 in the two archives. It shows that in 2015, before the publication of Jolie’s op-ed, average activity levels were almost three times higher than in 2013, with on average 197 tweets posted daily compared to 72. Moreover, Jolie’s second op-ed had a much weaker impact on the size of the BRCA Twitter stream than the 2013 one. In fact, on the day Jolie’s second op-ed was published, Twitter activity levels only rose to 1,805 daily tweets, while on the day her first op-ed was published, they reached a total of 7,965 tweets.

To study broadcasting and gatekeeping dynamics (RQ1), I examined the use of Twitter conversational markers, with social network analysis techniques being employed to identify prominent actors. In particular, measures of outdegree and indegree centrality (Freeman, 1979) were used to translate actor broadcasting ad gatekeeping prominence. In social network analysis, a node’s outdegree centrality measures how often that node acts as a sender of directed edges. A node’s indegree centrality weighs the number of directed edges that node receives from other nodes in the network. If we focus on Twitter as a networked platform, users can be interpreted as network nodes, while interactions via conversational markers (i.e., RT, @, and via) can be translated into directed edges between nodes. Outdegree centrality can then be used to measure how often a user retweets, mentions, or vias other users (i.e., user’s broadcasting prominence), while indegree centrality can be used to measure how often a user is retweeted, mentioned, or viaed by others (i.e., user’s gatekeeping prominence).

To investigate frame articulations (RQ2), I focused on hashtag co-occurrence networks. In hashtag co-occurrence networks, nodes stand for hashtags and undirected edges represent co-occurrences: an undirected edge between two nodes indicates that those nodes’ corresponding hashtags co-occurred in at least one tweet in the sample. I then used degree centrality (Freeman, 1979) measures—or each node’s likeliness to share an edge with any other node in the network—to visualize most frequently co-occurring hashtags.

Finally, to provide a comprehensive comparison of discursive practices and platform functionality for the BRCA issue public at the two time points being investigated (RQ3), the analytical phases discussed above were replicated over three time periods within each archive: (1) up until the day before the op-ed publication, (2) from the day of the op-ed publication until the last day with daily number of tweets higher than any day before the publication, and (3) in the remaining time in the sample period.

## Content Curation: Broadcasting and Gatekeeping Dynamics

Given its socio-technical infrastructure, Twitter hosts “intense curation practices” (Bennett et al., 2014, p. 245) that realize themselves via conversational markers. The use of RT, @, and via allows users to broadcast tweets before they disappear, crowdsourcing their authors to gatekeeping prominence. By mapping the use of RT, @, and via, the two following subsections will discuss broadcasting and gatekeeping dynamics in the 2013 and 2015 samples, respectively.

### 2013

Table 1 maps top broadcasters in the 2013 sample period, that is, users with highest outdegree scores or who most often used conversational markers (i.e., RT, @, and via). The three columns list broadcasters in the month before Jolie’s announcement (i.e., 14 April–13 May 2013), during the first 8 days after the piece’s publication, when the daily number of tweets was higher than ever before (i.e., 14 May–21 May 2013), and in the remaining period (i.e., 22 May–13 June 2013). Data show that, across the Twitter users in the top 10 broadcasting positions, different actors gained influence over time, with however US-based advocacy organization Breast Cancer Action (i.e., BCAction) recurring before, during, and after the publication of Jolie’s op-ed (bolded).^5^

**Table 1.** Top Broadcasters in the 2013 Sample Period.

	Before	During	After
1	FloridaForce	Shirakrance	AstleyClarke
2	Eperlste	Darwinianfail	Yablon
3	**BCAction**	Abcdiagnosis	**BCAction**, JoannaRudnick
4	Individual_6	Yablon	BRCAGeneAware
5	BRCAUmbrella	ABHuret	BRCAUmbrella
6	SLLitchy	FacingOurRisk	abcdiagnosis, PennMedicine, Pink_Hope
7	chemobrainfog	**BCAction**, PitzPoodle, Tealtoes	Individual_3
8	PinkMoonLovelie	CheckYourGenes, DrAttai, Individual_3	BRCAStudyBC, Jamesian, Kartemquin
9	individual_1, Tealtoes	Chemobrainfog	retnobi91
10	BRCAinfo	BRCAinfo	DrAttai, FloridaForce, GDM80, yale79DAV

The rankings are based on absolute outdegree values.

Even among broadcasters prominent across two of the three periods (underlined), advocacy organizations (i.e., abcd, BCCampaign, BRCAUmbrella, Florida Force and Kartemquin, and Tealtoes) played a primary role. But what was the result of these broadcasting dynamics, that is, which nodes in the conversational network were broadcasted to gatekeeping prominence?

**Figure 2.**
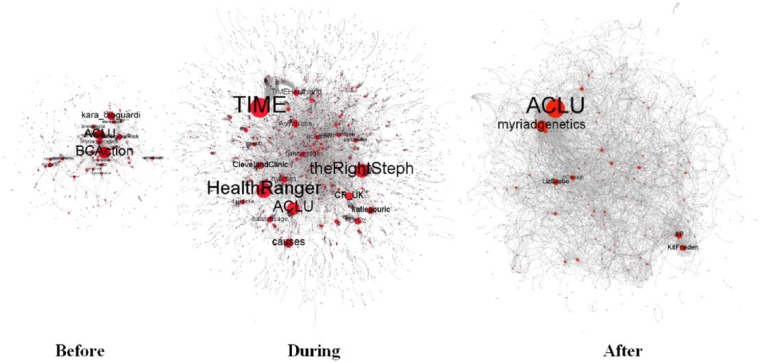
User interactions in the 2013 sample period (Gephi, graphs based on Forced Atlas algorithm).

Figure 2 maps the evolution of the BRCA Twitter conversational network over the 2013 sample period. Node and label size indicates indegree centrality, that is, the bigger the node, the more often its corresponding Twitter user was referenced via a conversational marker and stronger its gatekeeping power. The graphs show that the exposure of Jolie’s story, beyond boosting Twitter activity levels, temporarily decentralized gatekeeping power, that is, it increased the number of top gatekeepers in the first 8 days after the public announcement (“During” graph). Despite this, US-based legal and advocacy organization ACLU recurred as a top gatekeeper—and with a high gatekeeping score—across the three periods (bolded in Table 2), while US-based advocacy organizations Breast Cancer Action and Force (i.e., Facingourrisk) and pharmaceutical company Myriad Genetics (i.e, myriadgenetics) acted as prominent gatekeepers before and after the publication of Jolie’s op-ed. But let us have a closer look at the gatekeeping dynamics specifically developing in the three different time periods.

**Table 2.** Top Gatekeepers in the 2013 Sample Period.

	Before	During	After
1	BCAction	TIME	**ACLU**
2	**ACLU**	HealthRanger	myriadgenetics
3	kara_dioguardi	theRightSteph	AP
4	FacingOurRisk	**ACLU**	KitFrieden
5	myriadgenetics	causes	BCAction
6	sandrasparkly	CR_UK	LizSzabo
7	Individual_6	ClevelandClinic	FacingOurRisk
8	elizabethiorns	katiecouric	BCCampaign
9	mrgunn	DrOz	amyverner
10	ACLULive	AstroKatie	xeni

The rankings are based on absolute indegree values.

### Before

Before the public announcement, Breast Cancer Action, Force and Myriad Genetics acted as prominent gatekeepers, together with a few individual Twitter users. Myriad Genetics’ gatekeeping prominence before the public announcement is directly linked to events happening on the ground in relation to the ongoing judicial case “Association for Molecular Pathology versus Myriad Genetics,” where ACLU itself served as counsel for the plaintiffs. The case challenged the legitimacy of Myriad Genetics’ human gene patents and was specifically relevant to the BRCA Twitter stream as human gene patents increased the price of BRCA genetic testing. In fact, Myriad Genetics played a prominent role as a target of tweets campaigning against human gene patenting.

### During

In the conversational network developing during the first 8 days after the publication of Jolie’s op-ed (“During” graph in Figure 4), the ambient nature of Twitter conversational practices emerged with the coming to prominence of mainstream media outlets (i.e., Time) among advocacy organizations (i.e., CR_UK, or Cancer Research UK), individual advocates (i.e., HealthRanger) news editors (i.e., theRightSteph), and public figures (i.e., journalist Katie Couric and astrophysicist Katherine Mack, or AstroKatie). In fact, with the mediatization of Jolie’s BRCA narrative, traditional, elite gatekeepers temporarily entered the BRCA Twitter stream, combining with—but not replacing—non-elite ones, in a “hybrid news system” (Chadwick, 2013).

### After

Over the remaining period, Myriad Genetics and ACLU became by far the most prominent gatekeepers. On 13 June 2013, the US Supreme Court decided that naturally occurring DNA sequences, like BRCA1 and BRCA2, could no longer be patented. This is the reason behind the sudden rise in Twitter activity levels on 13 June 2013 (i.e., Day 61 in Figure 1) and behind the concentration of gatekeeping prominence on ACLU and Myriad Genetics in the “After” Twitter conversational network (Figure 2). The advocacy organizations in leading gatekeeping roles before the publication of Jolie’s op-ed (i.e., Facingourrisk and BCAction) also re-emerged here among a number of new individual Twitter users.

Overall, findings show that through Jolie’s first public announcement, health advocacy organizations were the primary curators (i.e., broadcasters and gatekeepers) of BRCA-related Twitter content. The BRCA stream was then mainly sourced via “organizationally enabled connective action” (Bennett & Segerberg, 2013), or via connective action mobilized by non-elite collective actors, that is, advocacy organizations. In line with recent work on the role of social media in rare disease advocacy (Vicari & Cappai, 2016), these findings also show that in 2013, Twitter was a communication and mobilization platform clearly embedded in the work of BRCA advocacy organizations. Jolie’s announcement had a short-term effect on curation dynamics: it temporarily introduced a hybrid dimension in the BRCA Twitter stream, with mainstream media sharing gatekeeping prominence with advocacy organizations and individual Twitter users.

### 2015

Data from the 2015 sample period indicate that in a 2-year span curation practices within the BRCA Twitter stream changed significantly. The three columns of Table 3 list top broadcasters in the month before Jolie’s announcement (i.e., 24 February–23 March 2015), during the first 4 days following the publication, when the daily number of tweets was higher than ever before (i.e., 24 March–27 March 2015), and in the remaining period (i.e., 28 March–23 April 2015). Data show that five out of the six top broadcasters who recurred before, during, and after Jolie’s announcement (bolded) were individual self-declared patient advocates mobilizing around BRCA conditions and hereditary cancer (i.e., BRCAresponder, karenBRCAMTL, BRCAinfo, and NickiDurlester) or Lynch Syndrome^6^ and hereditary cancer (i.e., ShewithLynch). Two out of the three broadcasters recurring in two of the three periods (underlined) were also breast cancer individual advocates (i.e., LguzzardiM, Individual_5).

**Table 3.** Top Broadcasters in the 2015 Sample Period.

	Before	During	After
1	**BRCAresponder**	**BRCAresponder**	**karenBRCAMTL**
2	**BRCAUmbrella**	**BRCAUmbrella**	**BRCAresponder**
3	**ShewithLynch**	**ShewithLynch**	**ShewithLynch**
4	**karenBRCAMTL**	DoveMed	**BRCAUmbrella**
5	Individual_2	**BRCAinfo, karenBRCAMTL**	Individual_5
6	OvarianCancerUK	Pinkandbluedoc	**BRCAinfo**
7	**BRCAinfo**	Individual_5	**NickiDurlester**
8	pinkandbluedoc	Tmskr401	LguzzardiM
9	**NickiDurlester**	**NickiDurlester**	Hc_chat
10	LguzzardiM	double_whammied	Individual_4, MHBTmovie

The rankings are based on absolute outdegree values.

**Figure 3.**
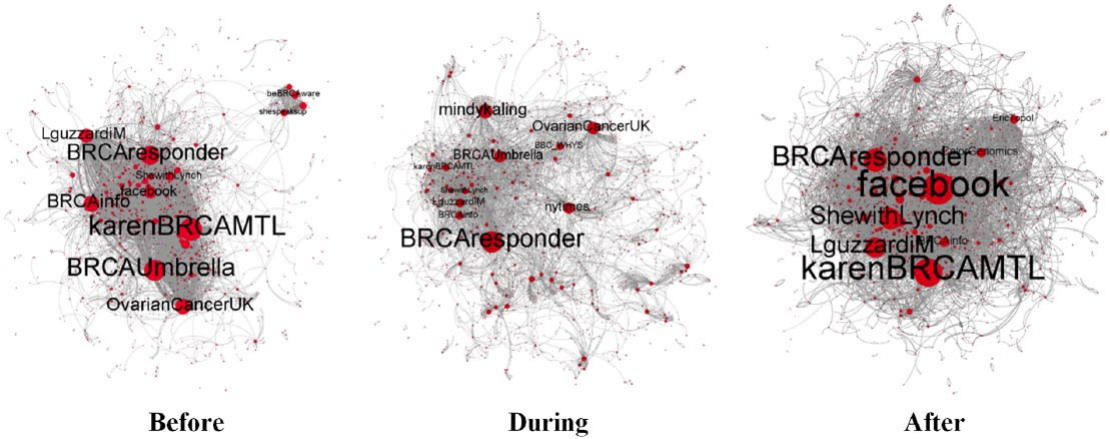
User interactions across the 2015 sample period (Gephi, graphs based on Forced Atlas algorithm).

Dynamics in the conversational network also varied compared to 2013 (Figure 3). The graphs in Figure 3 show that gatekeeping power was shared by more nodes before and after the exposure of Jolie’s story than during the exposure itself. This suggests that at times of non-heightened mainstream media coverage, more actors had consolidated gatekeeping prominence than in 2013.

In fact, Table 4 shows that five individual self-declared patient advocates (i.e., karenBRCAMTL, BRCAresponder, BRCAinfo, Lguzzardi, and ShewithLynch, bolded in Table 4) recurred as top gatekeepers over the entire sample period. But let us focus more specifically on the gatekeeping dynamics emerging in the three different time periods.

**Table 4.** Top Gatekeepers in the 2015 Sample Period.

	Before	During	After
1	**karenBRCAMTL**	**BRCAresponder**	facebook
2	BRCAUmbrella	mindykaling	**karenBRCAMTL**
3	**BRCAresponder**	OvarianCancerUK	**BRCAresponder**
4	OvarianCancerUK	BRCAUmbrella	**ShewithLynch**
5	**BRCAinfo**	nytimes	**LguzzardiM**
6	**LguzzardiM**	**LguzzardiM**	**BRCAinfo**
7	facebook	**BRCAinfo**	ColorGenomics
8	**ShewithLynch**	**karenBRCAMTL**	pinkandbluedoc
9	ClairaHermet	BBC_WHYS	MyGeneCounsel
10	beBRCAware	**ShewithLynch**	EricTopol

The rankings are based on absolute indegree values.

### Before

Before the exposure of Jolie’s story, several individual patient advocates (e.g., karenBRCAMTL, BRCAresponder, BRCAinfo, LguzzardiM, and ShewithLynch) played top gatekeeping roles along with only two advocacy organizations (i.e., BRCAUmbrella and OvarianCancerUK). Facebook was among the top mentioned sources, primarily due to a high number of tweets campaigning against the removal of mastectomy photos on BRCA Facebook pages.

### During

Like in 2013, with the publication of Jolie’s op-ed, traditional news outlets (i.e., *New York Times* and BBC) temporarily reached gatekeeping prominence (see “During” graph in Figure 3). They populated a highly hybrid gatekeeping system along with US celebrity Mindy Kaling, advocacy organizations Ovarian Cancer UK, and the scientific journal *JAMA*.

### After

During the remaining period, individual patient advocates (e.g., karenBRCAMTL, BRCAresponder, ShewithLynch, LguzzardiM, BRCAinfo, and MyGeneCounsel) played an even stronger gatekeeping role than before the publication of Jolie’s piece, with Facebook re-emerging as the target of campaigning against the removal of mastectomy photos on BRCA Facebook pages.

Overall, these results show a dramatic shift in Twitter curation practices occurring between 2013 and 2015, with individual patient advocates replacing advocacy organizations as top curators—in broadcasting and gatekeeping roles—of the BRCA Twitter stream. This change in curation practices then shows that in a 2-year span, connective action sourcing the BRCA Twitter stream went from being primarily “organizationally enabled” to essentially “crowd-enabled” (Bennett & Segerberg, 2013). Both Jolie’s announcements added a hybrid dimension in gatekeeping dynamics, but did not inhibit the role of pre-existing curators. We may speculate that this shift in connective action depends on both cultural and socio-technical factors. On one side, it is likely that a longitudinal rise in public awareness around BRCA-related topics—probably also linked to the exposure of Jolie’s story—led to the progressive emergence of personalized forms of engagement with them. On the other side, the manifestation of these personalized forms of engagement was enhanced by Twitter’s socio-technical infrastructure that allowed non-elite *individual* actors (i.e., patient advocates) to engage in content curation processes that 2 years earlier were controlled by non-elite *collective* actors (i.e., advocacy organizations). In other words, these findings suggest that not only can Twitter broadcast networks open up space for minorities’ viewpoints (Benkler, 2006; Jackson & Foucault Welles, 2015, 2016), they can also allow significant power shifts between collective and individual actors within minorities themselves.

## BRCA Framing: Dominant Narratives and Meaning Construction

Twitter curation dynamics, or interactions between broadcasters and gatekeepers, lead to the emergence of dominant narratives—or frames—that work “through persistent patterns of selection, interpretation, emphasis, exclusion, and retention” (Meraz & Papacharissi, 2013, p. 143). Frames, as composite cognitive elements, develop via the use of language-specific devices like Twitter’s # symbol. Hashtags segment broad issue publics—like the BRCA public—around topic-specific streams. By looking at hashtag co-occurrence networks, or the combined use of different hashtags within a Twitter stream, it is then possible to map the articulation of broad issues along topical dimensions and identify dominant narratives associated with them. The two following subsections will discuss framing dynamics in the 2013 and 2015 sample, respectively.

### 2013

Table 5 maps the top hashtag co-occurrences in the 2013 sample period and shows that the only hashtag pair recurring over the whole period (bolded) associated the BRCA gene mutation with breast cancer.

**Table 5.** Hashtag Pairs with Top 10 Frequencies over the 2013 Sample Period.

	Before		During		After	
	Hashtag 1	Hashtag 2	Hashtag 1	Hashtag 2	Hashtag 1	Hashtag 2
1	BRCA	SCOTUS	**BRCA**	**breastcancer**	BRCA	SCOTUS
2	**BRCA**	**breastcancer**	BRCA	AngelinaJolie	**BRCA**	**breastcancer**
3	BRCA	genepatent	BRCA1	cancer	BRCA	genes
4	BRCA	genepatents	BRCA1	breastcancer	SCOTUS	genes
5	BRCA	cancer	BRCA1	AngelinaJolie	SCOTUS	Myriad
6	BRCA SCOTUS	Myriad breastcancer	BRCA1	breast	BRCA	BCSM
7	BRCA	hgprally	BRCA	BCSM	BRCA BCSM	genepatents breastcancer
8	SCOTUS SCOTUS	genepatents genepatent	BRCA	mastectomy	BRCA	Myriad
9	BRCA	previvor	BRCA	cancer	SCOTUS	breastcancer
10	SCOTUS	hgprally	breastcancer	AngelinaJolie	genepatents	DNA

**Figure 4.**
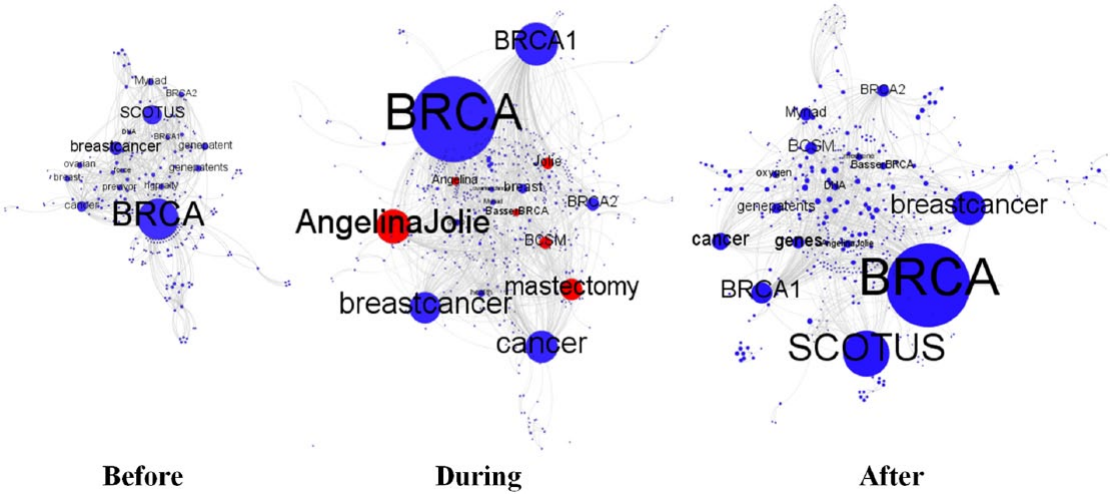
Hashtag networks across the 2013 sample period (Gephi, graphs based on Forced Atlas algorithm).

Figure 4 maps hashtag co-occurrence networks across the publication of Jolie’s first op-ed. Nodes stand for hashtags, while the presence of an edge between two nodes indicates that those nodes’ corresponding hashtags co-occurred in at least one tweet. Node and label size indicates degree centrality: the bigger a node, the more often its corresponding hashtag was used in tweets also containing other hashtags. Red nodes stand for prominent hashtags originating with Jolie’s announcement.

### Before

The top hashtag pairs used before the publication of Jolie’s op-ed show that before heightened mainstream media exposure, the BRCA gene mutation was primarily discussed in terms of gene patents and their implications. The corresponding hashtag network shows that the BRCA Twitter stream was almost entirely focused on Myriad Genetics’ ownership of the BRCA gene patents, with attention being drawn to the ongoing legal case. #hgprally—for instance, that in the first hashtag network frequently co-occurred with the top hashtags #BRCA and #SCOTUS (i.e., Supreme Court of the United States)—was an event hashtag used during the Human gene patent rally of 15 April 2013. The rally, organized by Breast Cancer Action, took place on the steps of the US Supreme Court while arguments against gene patents were being presented to the Court (Carmody and Sartor, 2013). Hashtags like #gene, #genepatents, #genepatent, and #previvor were also frequently linked to #BRCA and #SCOTUS.

### During

Jolie’s announcement had short-term effects, the most evident of which being a sudden shift toward Jolie’s BRCA-related narrative, with the foregrounding of the BRCA1 gene mutation over the BRCA2 gene mutation^7^ and of prophylactic mastectomy as a preventive measure. Finally, Jolie’s story enhanced the emergence of dedicated Twitter chats (i.e., BCSM).

### After

In the remaining period, top hashtag pairs reframed the BRCA issue stream around genes, gene patents, and the trial against Myriad Genetics like in the period prior to the publication of Jolie’s editorial, indicating that the Angelina effect on framing dynamics was extremely short-termed.

These results show that across the publication of Jolie’s first op-ed, the BRCA Twitter stream developed around both content-based frames descriptive of real-world events on the ground and more volatile and short-lived content-based frames emerging with Jolie’s story. Hence, Twitter functioned primarily as a “news reporting mechanism” (Papacharissi & de Fatima Oliveira, 2012), hosting, for instance, the live coverage of the Human gene patent rally but also enhancing the inclusion of Jolie-related BRCA articulations. The co-presence of both framing dynamics suggests that users engaged in connective action with different levels of agency, with Twitter having both mobilizing (Bennett & Segerberg, 2013) and ambient (Hermida, 2010; Bruns & Burgess, 2012) functions.

### 2015

Table 6 lists the top hashtag pairs over the 2015 sample period. Data show that in 2015 not only was the BRCA gene mutation regularly associated with breast cancer—like in 2013—it was also constantly linked to the hashtag #BCSM, tagging a breast cancer Twitter chat (bolded).

**Table 6.** Hashtag Pairs with Top 10 Frequencies over the 2015 Sample Period.

	Before		During		After	
	Hashtag 1	Hashtag 2	Hashtag 1	Hashtag 2	Hashtag 1	Hashtag 2
1	**BRCA**	**BCSM**	BRCA	AngelinaJolie	**BRCA**	**BCSM**
2	**BRCA**	**breastcancer**	BRCA	cancer	BCSM	breastcancer
3	BCSM	breastcancer	BRCA	ovariancancer	mastectomy	breastcancer
4	BRCA	ovariancancer	**BRCA**	**breastcancer**	**BRCA**	**breastcancer**
5	BRCA	mastectomy	BRCA	HCChat	BCSM	mastectomy
6	mastectomy	breastcancer	BRCA	hereditarycancer	BRCA	mastectomy
7	BCSM	mastectomy	**BRCA**	**BCSM**	BRCA	facebook
8	BRCA	bckills	cancer	AngelinaJolie	mastectomy	facebook
9	BRCA metsmonday metsmonday	Lynchsyndrome bckills dontignorestageiv	AngelinaJolie	ovariancancer	BCSM breastcancer BCSM BRCA breastcancer facebook	facebook facebook photos photos photos photos
10	BRCA	facebook	BRCA	HCChat	BRCA	Lynchsyndrome

**Figure 5.**
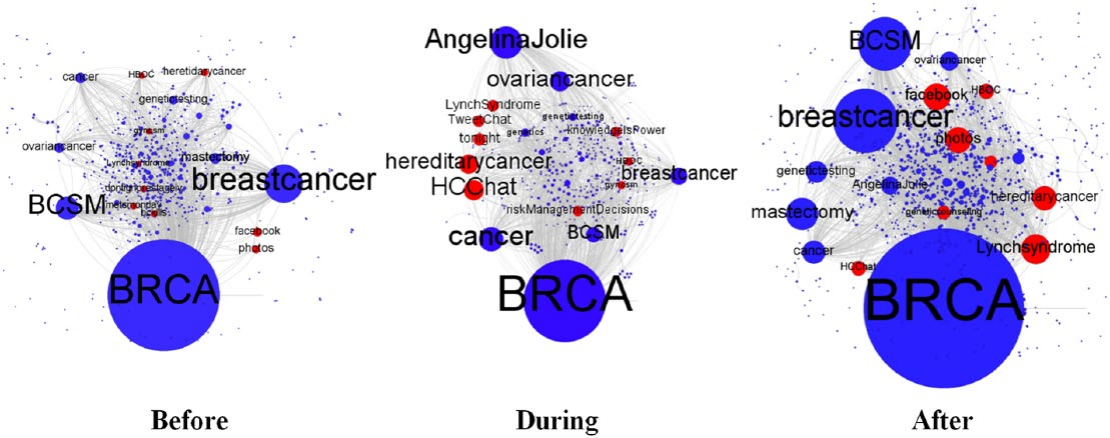
Hashtag networks across the 2015 sample period (Gephi, graphs based on Forced Atlas algorithm).

Figure 5 maps the complete networks of co-occurring hashtags across the publication of Jolie’s second op-ed. Red nodes stand for new prominent hashtags compared to 2013.

### Before

Looking at both Table 6 and Figure 5, it is evident that before Jolie’s announcement, the BRCA Twitter stream was mainly developing around breast cancer narratives. But while mastectomy was a popular co-occurring hashtag already in 2013, the new frequent use of the hashtag #metsmonday—tagging a Monday chat for people with metastatic cancer—with #dontignorestageiv and #bckills (see Table 6) signals the emergence of new framing dynamics. It indicates a turn in the discussion toward more specialist themes associated with breast cancer, themes more popular within the environmental—or political—breast cancer movement (Klawiter, 1999; McCormick, Brown, & Zavestoski, 2003) than in mainstream representations of breast cancer. This specialist turn is also signaled by the frequent association of BRCA with Lynch Syndrome, as this association indicates awareness of similarities between the BRCA gene mutation with other hereditary cancer conditions (e.g., Lynch Syndrome). These findings suggest the appropriation of the BRCA Twitter stream by activist actors and a consequent shift toward the emergence of activist action frames. In fact, these framing dynamics suggest that by 2015, the BRCA Twitter stream had expanded its scope as an awareness system, hosting more dynamic articulations of BRCA-related topics than in 2013.

### During

In the short term, Jolie’s announcement—like in 2013—mainly introduced frame articulations directly linked to Jolie’s BRCA narrative. First, it drew attention to the hereditary nature of the BRCA gene mutation via the use of #BRCA with #HCChat—a Twitter chat on hereditary cancer—and #hereditarycancer. Second, it foregrounded inspirational hashtags like #knowledgeispower. Third, it drew attention to ovarian cancer–related narratives via hashtags like #HBOC—tagging hereditary breast and ovarian cancer tweets—and #gyncsm, a Twitter chat for individuals impacted by gynecological cancers.

### After

Finally, dynamics similar to those described for the period before the publication of Jolie’s op-ed are evident in the hashtag network developing toward the end of the 2015 sample period. There, #mastectomy, #breastcancer, and #BCSM are frequently associated with #BRCA, and Lynch Syndrome is again linked to the BRCA gene mutation. In this hashtag network, #facebook also gains visibility due to the ongoing campaign against Facebook’s removal of mastectomy photos described in the analysis of curation dynamics.

Overall, these framing dynamics suggest that while in April–June 2013 the BRCA Twitter stream primarily functioned as a news reporting mechanism centered around content-based frames, in February–April 2015, it worked more as an “awareness system” (Hermida, 2010) where narratives developed around specialist topical dimensions. In both sample periods, the exposure of Jolie’s story temporarily introduced frame articulations directly linked to Jolie’s BRCA narrative. These findings, coupled with previous results on shifting curation practices, picture the BRCA Twitter stream as developing—between 2013 and 2015—via the foregrounding of personalized over collective forms of engagement, with patient advocates emerging as top curators of specialist narratives of health and illness.

## Discussion and Conclusion

Empirical work interested in the impact of social media usage on discursive practices in general—and public sphere dynamics in particular—has drawn attention to the way social media’s socio-technical infrastructures tend to destabilize traditional power roles in the construction of meaning around issues of public interest. When it comes to Twitter, several studies have investigated one key time point in the life story of a Twitter stream, unveiling the platform’s democratizing potential in allowing non-elite actors to come to prominence in both the selection of information sources and the construction of meaning around specific issues. What is generally missing in previous research is a focus on the temporal dimension of discursive practices, that is, how Twitter functionality for an issue public may differ at different time points in the life story of an issue-based Twitter stream.

Drawing upon recent research on Twitter discursive practices, this article investigated curation and framing dynamics at two similar but temporarily distant time points in the life story of the BRCA Twitter stream. The ultimate goal was to potentially unveil changes in platform functionality, that is, changes in the way Twitter usage allowed or supported the coming to prominence of non-elite actors described in previous research (Bastos et al., 2013; Hermida, 2015; Jackson & Foucault Welles, 2016; Meraz & Papacharissi, 2013).

This study’s findings on broadcasting and gatekeeping dynamics (RQ1) showed that curation patterns at any time point in an issue-based Twitter stream reflect transient power structures. In the BRCA Twitter stream, between 2013 and 2015, individual patient advocates supplanted advocacy organizations in top broadcasting and gatekeeping roles, with crowd-enabled replacing organizationally enabled connective action (Bennett & Segerberg, 2013). In other words, not only did Twitter usage enhance the emergence of non-elite actors (Jackson & Foucault Welles, 2016; Meraz & Papacharissi, 2013) in the curation of BRCA topics, it also allowed the transfer of power roles from non-elite *collective* actors (i.e., advocacy organizations) to non-elite *individual* actors (i.e., patient advocates), that is, to minorities within minorities.

Findings also show that frame articulation (RQ2) can highly vary at different time points of a Twitter stream. In 2013, the BRCA gene mutation was primarily discussed via content-based frames relevant to Myriad Genetics’ gene patents controversy. By February 2015, the BRCA stream had developed specialist narratives, not only foregrounding breast cancer themes hardly present in mainstream representations of breast cancer (e.g., metastatic cancer) but also drawing links with similar but far less known hereditary cancer conditions (i.e., Lynch Syndrome). This highlights the need to investigate issue involvement not only in terms of its discursive articulations (e.g., frames) but also as situated in socio-technical spaces where meaning is linked to actors (e.g., tweeters), technology (e.g., Twitter), and events (e.g., sudden mainstream media exposures) (Marres, 2007).

What remained unvaried in the BRCA stream between 2013 and 2015 was the temporary effect of heightened mainstream media exposure linked to Angelina Jolie’s *New York Times* opinion pieces. In both occasions, the “Angelina effect” added a “hybrid” (Chadwick, 2013) dimension to the BRCA Twitter stream—with mainstream media sharing gatekeeping prominence with non-elite actors,—bolstered the emergence of BRCA-themed Twitter chats and foregrounded BRCA-related topics directly linked to the actress’ experience of the BRCA condition. The hybrid dimension caused by the Angelina effect was however short-lived, with curation and framing dynamics occurring prior to Jolie’s pieces resuming a few days after their publication.

Ultimately, this study indicates that—while dynamics of media hybridization show elements of stability—Twitter’s overall functionality (RQ3) for non-elites within an issue public is highly fluid. For the BRCA public, for instance, in April–June 2013, the platform worked primarily as an organization-centered “news reporting mechanism” (Papacharissi & de Fatima Oliveira, 2012), focusing on events happening on the ground. In February–April 2015, these dynamics had been overturned by the emergence of individual actors—that is, patient advocates—curating BRCA-related content along specialist topical dimensions. In this second scenario, Twitter worked then more as a crowdsourced specialist “awareness system” (Hermida, 2010, p. 298).

These findings suggest that the effects of new socio-technical infrastructures on the expression of non-elite actors combine with wider cultural dynamics, that is, the coming to prominence on social media of non-elite actors may vary according to—non-platform bound—cultural dynamics. In the case of the BRCA issue public, for instance, rising public awareness around BRCA-related topics—most likely also linked to the “Angelina effect”—led to the development of personalized forms of engagement with these topics. The contemporary emergence of “active patients” described in previous research (Vicari & Cappai, 2016, p. 1655) probably also provided a fertile background for the development of personalized forms of engagement with BRCA-related topics. Twitter’s socio-technical infrastructure, in particular its hosting specific networked curation and framing processes regulated by conversational and tagging markers, simply enhanced the *public* manifestation of these personalized forms of engagement. Hence, in a 2-year span, as a result of the combination of non-platform bound cultural dynamics and platform-specific discursive practices, the public voice of individual patient advocates in the BRCA Twitter stream overcame that of traditional collective advocacy actors.

This article points at Twitter’s fluid functionality and suggests that by looking at the life story—rather than at a single time point—of an issue-based Twitter stream, we can better understand the contribution of new socio-technical infrastructures as embedded in wider cultural dynamics. In particular, we can map the development of power roles in discursive practices and understand the dynamics underlying the emergence of non-elite actors in the public arena. Finally, this article provides evidence of the rise of activist cultures that rely on fluid, non-elite, collective, and individual social media engagement.

## Notes

1. The BRCA gene mutation—BRCA1 and BRCA2—is a rare hereditary genetic condition that causes an increased risk to develop breast and ovarian cancer (National Cancer Institute, 2017).

2. Meraz and Papacharissi (2013) use the similar term “networked social awareness systems.”

3. For cross-disciplinary, pivotal discussions of frame as a theoretical concept and a unit of analysis, see, among the others, Entman (1993), Gitlin (1980), Snow et al. (1986), and Tuchman (1978).

4. Cleaning was a two-step process. First, having browsed originally retrieved entries, I developed ad hoc queries on Discovertext to filter out non-relevant tweets. Second, I manually checked the remaining entries to exclude non-relevant tweets that had not been filtered out in the previous step.

5. The article reports original Twitter handles of organizations, public figures, journalists, news editors, individuals who have publicly spoken of their engagement with BRCA advocacy outside Twitter or individuals who have given their consent to disclose their Twitter handle in this study. Where none of these conditions was met, Twitter handles were replaced with pseudonyms.

6. Lynch Syndrome is an inherited cancer syndrome derived from a genetic mutation that—like BRCA1 and BRCA2—increases the risk of different types of cancer at a young age (Genetic and Rare Diseases Information Center, 2016).

7. In her 2013 op-ed, Angelina Jolie explained that her inherited gene mutation was BRCA1.
